# High-throughput analysis of the satellitome revealed enormous diversity of satellite DNAs in the neo-Y chromosome of the cricket *Eneoptera surinamensis*

**DOI:** 10.1038/s41598-017-06822-8

**Published:** 2017-07-25

**Authors:** Octavio Manuel Palacios-Gimenez, Guilherme Borges Dias, Leonardo Gomes de Lima, Gustavo Campos e Silva Kuhn, Érica Ramos, Cesar Martins, Diogo Cavalcanti Cabral-de-Mello

**Affiliations:** 1 UNESP - Univ Estadual Paulista, Instituto de Biociências/IB, Departamento de Biologia, Rio Claro, São Paulo Brazil; 2 Departamento de Biologia Geral, Universidade Federal de Minas Gerais, Belo Horizonte, MG Brazil; 3 UNESP - Univ Estadual Paulista, Instituto de Biociências/IB, Departamento de Morfologia, Botucatu, São Paulo Brazil

## Abstract

Satellite DNAs (satDNAs) constitute large portion of eukaryote genomes, comprising non-protein-coding sequences tandemly repeated. They are mostly found in heterochromatic regions of chromosomes such as around centromere or near telomeres, in intercalary heterochromatin, and often in non-recombining segments of sex chromosomes. We examined the satellitome in the cricket *Eneoptera surinamensis* (2n = 9, neo-X_1_X_2_Y, males) to characterize the molecular evolution of its neo-sex chromosomes. To achieve this, we analyzed illumina reads using graph-based clustering and complementary analyses. We found an unusually high number of 45 families of satDNAs, ranging from 4 bp to 517 bp, accounting for about 14% of the genome and showing different modular structures and high diversity of arrays. FISH mapping revealed that satDNAs are located mostly in C-positive pericentromeric regions of the chromosomes. SatDNAs enrichment was also observed in the neo-sex chromosomes in comparison to autosomes. Especially astonishing accumulation of satDNAs loci was found in the highly differentiated neo-Y, including 39 satDNAs over-represented in this chromosome, which is the greatest satDNAs diversity yet reported for sex chromosomes. Our results suggest possible involvement of satDNAs in genome increasing and in molecular differentiation of the neo-sex chromosomes in this species, contributing to the understanding of sex chromosome composition and evolution in Orthoptera.

## Introduction

Among the repetitive sequences in the genomes of eukaryotes, tandem repeats (TRs) are very abundant and are mostly represented by satellite DNAs (satDNAs). SatDNA sequences are mainly located in centromeric, telomeric or intercalary heterochromatin^1–3^ but, in some cases, also dispersed in euchromatin^4, 5^. This genomic fraction is composed of hundreds to thousands of noncoding tandemly-arrayed sequences with late-replication, and oriented in a head-to-tail fashion^1, 3, 6–9^.

SatDNA families, in general, differ in sequence identity, copy number and chromosome distribution^2, 10–12^. These sequences are subject to intragenomic concerted evolution, resulting in more efficient homogenization of repeats within species than between species and also between repeats located in the same array/chromosome than between different ones^2–4, 7^. Concerted evolution is achieved through multiple mechanisms of non-reciprocal transfer such as unequal cross-over, gene conversion, rolling-circle replication and transposition^13, 14^.

Sex chromosomes have arisen independently several times in a wide range of animals and plants from an ordinary autosomal pair^15, 16^, presenting as a recurrent trait the suppression of recombination and accumulation of distinct classes of repetitive DNAs, including satDNAs^1, 17–21^. In Orthoptera, the X0♂/XX♀ sex-determining system is considered modal^22, 23^ but eventually, diverse sex chromosome systems evolved several times, such as neo-XY♂/XX♀^24–26^, X_1_X_2_0♂/X_1_X_1_X_2_X_2_♀^27^ and even neo-X_1_X_2_Y♂/X_1_X_1_X_2_X_2_♀^25, 28^. It was found that particularly centric fusions (i.e. Robertsonian translocations) and tandem fusions with autosomes, dissociations and inversions contributed to the formation of neo-sex chromosomes in Orthoptera ^22, 23, 25–28^. The DNA composition of the orhtopteran neo-sex chromosomes was studied only in a few species, mainly focusing on certain types of repetitive DNAs, such as multigene families, C_*0*_ *t* DNA fraction, telomeric repeats and microsatellites arrays^25–29^.

Chromosomal evolution and repetitive DNA organization was addressed in the cricket *Eneoptera surinamensis*, a species with the genome size of 5.42 Gbp, chromosome number of 2n = 9♂/10♀ and a neo-X_1_X_2_Y♂/X_1_X_1_X_2_X_2_♀ sex-determining system^28^. During male meiosis the multiple sex chromosomes remain unpaired and do not form chiasmata, suggesting that they do not proceed recombination^22, 23^. The neo-Y chromosome is the largest element and it exhibits multiple heterochromatic bands, while the neo-X_1_ and neo-X_2_ are poor in C-positive heterochromatin. Of the repetitive DNAs, two multigene families (5S rDNA and histone genes), C_*0*_ *t* DNA fraction, and diverse microsatellites mapped to the neo-Y^28^.

In the present study, in order to provide comprehensive information on repetitive DNAs that occur in the genome, and specifically in the neo-sex chromosomes of *E*. *surinamensis*, we performed a detailed analysis of satDNAs (the satellitome)^30^, integrating genomic and chromosomal data. Our results revealed accumulation of satDNAs, with the occurrence of unusually high number of 45 distinct families in the *E*. *surinamensis* genome. In addition, some of these families were enriched in the highly differentiated neo-X_1_X_2_Y sex chromosomes in comparison to autosomes, mostly over-represented in the neo-Y. To our knowledge, this is the largest diversity of satDNAs yet reported for a Y chromosome in eukaryotes.

## Results

### Identification of satDNAs and sequence characterization

**Figure 1:**
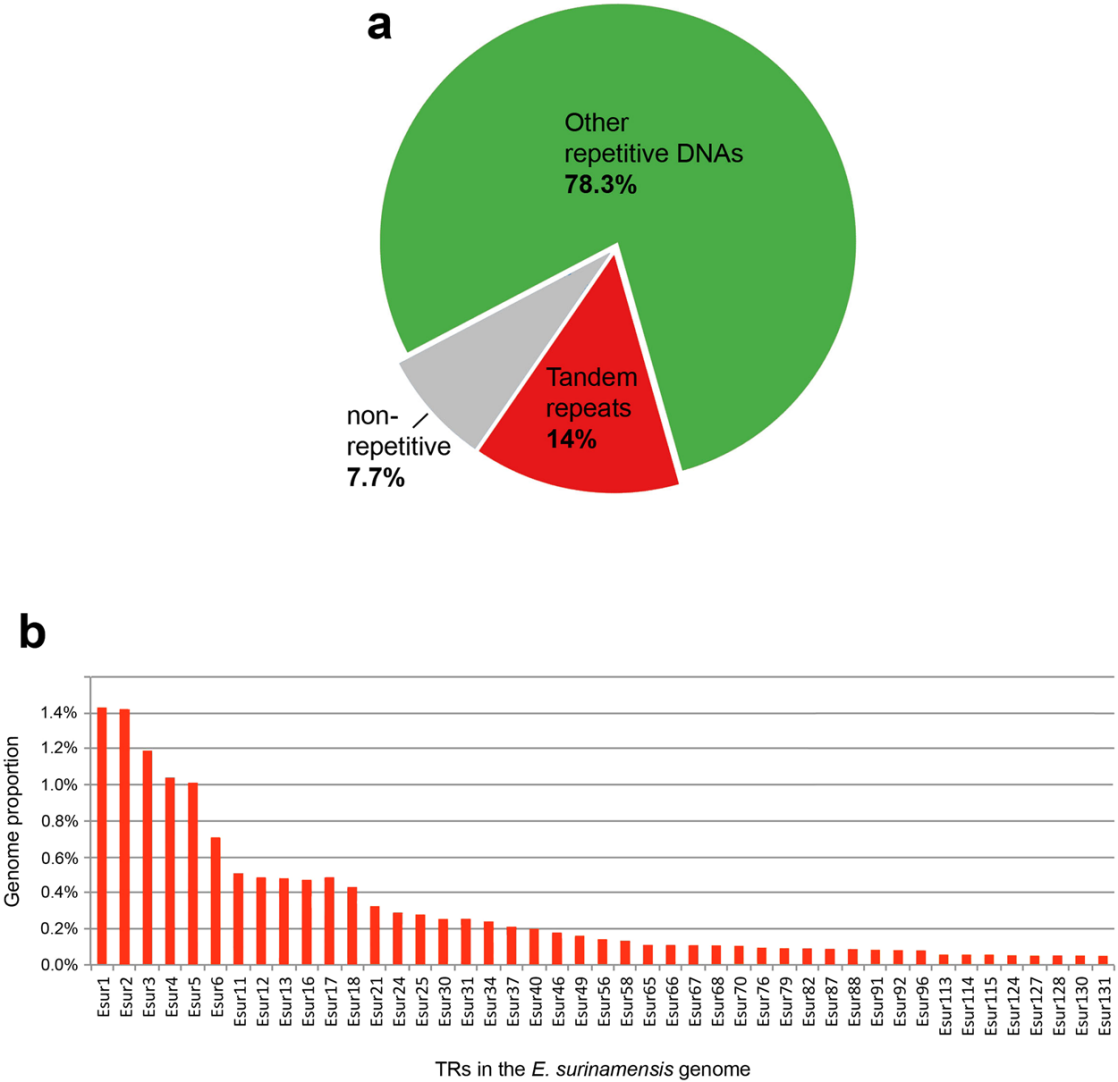
(**a**) Overview of the repetitive composition of *E*. *surinamensis* genome based on the output of RepeatExplorer. (**b**) Genomic proportion for each of the 45 satDNAs isolated and characterized. TRs, tandem repeats.

Illumina sequencing returned 5,872,912 paired-end reads (ranging from 35 to 288 nt, mean reads length of 164.6 nt) totaling 615,581,150 nt that were trimmed to 150 nt. Given the estimated genome size of 5.42 Gbp for *E*. *surinamensis* ^28^, this represents about 0.16× genome coverage. The clustering analysis through the RepeatExplorer^31, 32^ used as input 1,299,110 illumina previously trimmed paired-ends reads and produced 292,070 clusters (containing 92.3% of reads) differing in size, sequence composition and genomic abundance, including satDNAs and other non-characterized repetitive elements. The singletons should represent the low copy number fraction of the genome, which yielded in 100,002 singlets (containing 7.7% of reads) (Fig. 1a). A set of 131 of the most abundant clusters representing repetitive elements was analyzed in the search for satDNAs. A representation of the genomic proportion of 45 clusters identified as satDNAs is showed in Fig. 1b.

The analysis of dotplots confirmed the occurrence of 45 families of tandem repeats ranging from 4 to 517 bp long, showing different modular structure and array diversity. Among them, 21 satDNAs families were recovered through PCR that revealed a ladder pattern. For the remaining repeat families, with small monomers (less than 60 bp), we were not able to confirm the ladder pattern through PCR due to the very limited window for primer design and amplification. The characteristics for all satDNAs families are presented in the Supplementary Tables S1 and S2. Altogether, the 45 satDNAs families comprised about 14% of the male genome that represent 758,8 Mb of 5,420,00 Mb of species genome size. The nucleotide divergence within the families varied from 1.9 to 30.2%. The nucleotide sequences showed an A + T content ranging from 15 to 67.15%. It can be concluded that most of the satDNAs analyzed here constitute light satDNAs due to the low A + T content (see Supplementary Tables S1 and S2).

For two satDNAs, i.e. Esur17 and Esur18, two subfamilies were identified showing an individual repeat called α and a composite repeat called α/β (see Supplementary Table S2 and Supplementary Fig. S1). There was no significant sequence similarity between α and β sequences. The size of the Esur17 α-repeat was 163 bp while the Esur17 α/β composite repeat was 317 bp, each consisting of an α-unit and additional 154 bp β-unit. The nucleotide divergence between both Esur17-α and Esur17-α/β repeats was 9.4% (see Supplementary Table S2). Regarding Esur18, our sequences analysis showed that this is an array composed of α/β composite units with 517 bp, consisting of a 329 bp α-unit and a 188 bp β-unit (see Supplementary Fig. S1). The Esur18 α/β repeats showed 4.3% nucleotide divergence (see Supplementary Table S2). We also detected sequence similarity between Esur2 and Esur34 repeats (~82.6%) and between Esur3 and Esur58 repeats (80.9%). This result explains the overlapping chromosomal distribution of both satDNA pairs (see below) and indicate that they are representatives of two satDNA families, Esur2/Esur34 and Esur3/Esur58. NJ trees showed Esur2, Esur34, Esur3 and Esur58 allocated in cluster-specific branches, indicating that each subfamily is composed of exclusive repeat-variants (see Supplementary Fig. S2). Both NCBI BLAST and Repbase searches, with the consensus monomer sequence belonging to each repeat family as a query, did not revealed significant similarity with any other previously described sequences.

Considering all satDNAs identified, we found that monomer size and nucleotide divergence displayed a somewhat low but significant negative correlation (*rho* = −0.48, *P* = <0.001). Also, monomer size and a total number of loci displayed a moderate negative correlation (*rho* = −0.63, *P* = <0.0001) (Supplementary Fig. S3).

### Chromosomal localization of satDNAs

**Figure 2:**
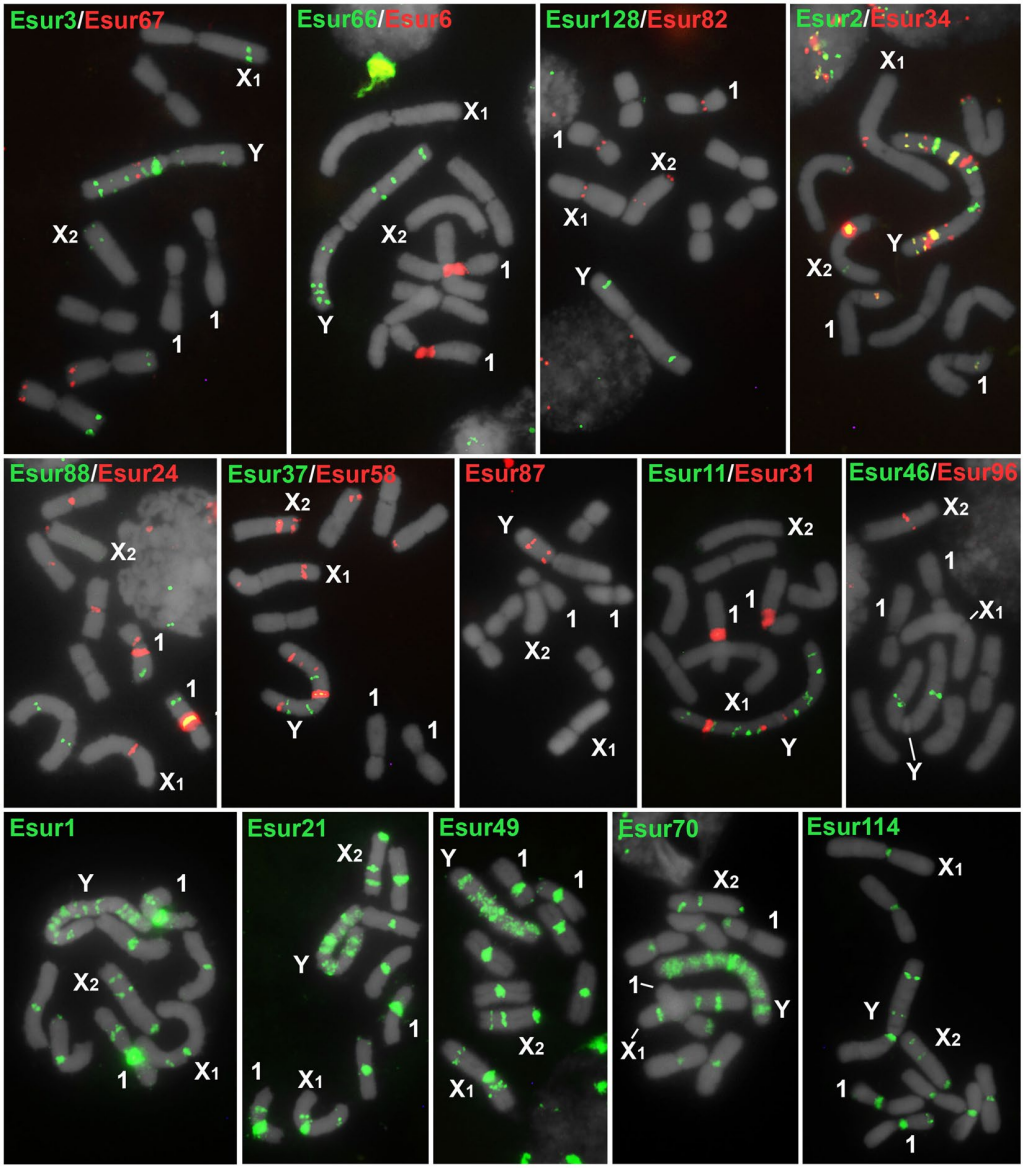
Chromosomal location of 22 satDNAs in mitotic chromosomes of male embryos of *E*. *surinamensis* by FISH. Upper and middle panels show satDNAs with monomers >60 bp, lower panels satDNAs with monomers <60 bp. The satDNA family names and hybridization signals for each type of the probe are shown in the images by colors. Note the enrichment in the neo-Y (Y) sex chromosome with multiple loci or spread signals for some repeats, while the X_1_ and X_2_ sex chromosomes show only a few or even none hybridization signals.

To detect the chromosomal localization of the 45 satDNA families single- or two-color FISH were carried out on male mitotic metaphases. In autosomes, distinct satDNAs were located mostly in the pericentromeric regions that correspond to the C-band positive blocks observed by Palacios-Gimenez *et al*.^28^. A few satDNA loci were also found in interstitial and distal C-band negative blocks. The patterns were variable depending on the repeat mapped (Fig. 2, Supplementary Tables S1 and S2). Most satDNAs were located in multiples autosomes, while some of them were located exclusively in one chromosome pair, for example, Esur4, Esur6, and Esur18- α/β, were all co-located in the centromere and secondary constriction of pair 1. Two-color fiber-FISH confirmed regions of interspersion between Esur4, Esur6 and Esur18- α/β repeats (see Supplementary Fig. S4). For Esur17, the α and α/β repeats were located in the secondary constriction of pair 1 and in the X_2_ chromosome; for Esur18, the α and β repeats were located in secondary constriction of pair 1, but additional multiple loci of the α repeats were detected in the Y chromosome (see Supplementary Table S2 and Supplementary Fig. S5).

**Figure 3:**
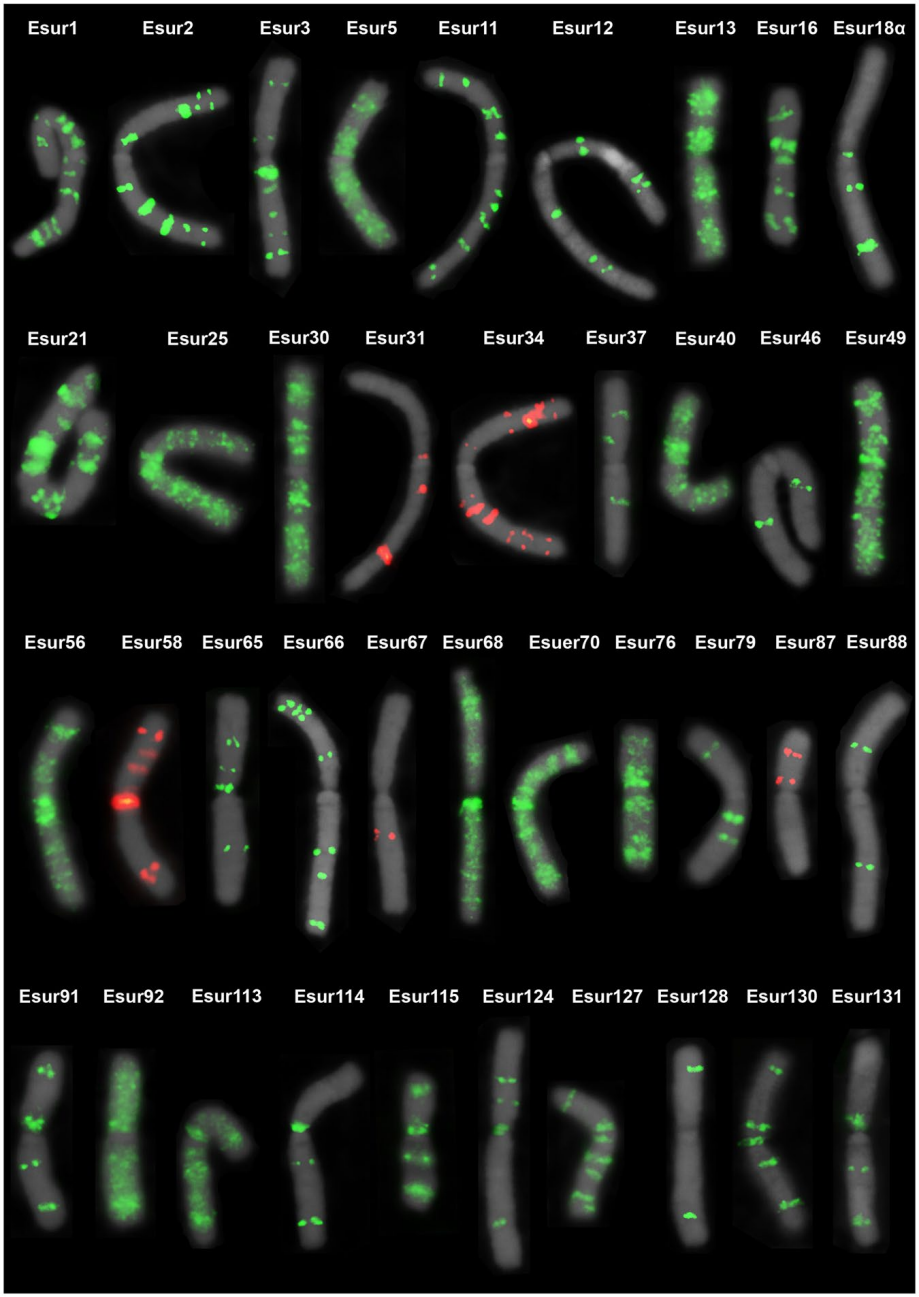
Distribution of the 39 satDNAs in the neo-Y chromosome of *E*. *surinamensis*.

Some of satDNAs families were placed in the neo-X_1_ and neo-X_2_ but the abundance (number of loci) of satDNAs on the neo-Y chromosomes was remarkable, showing either multiple discrete loci or a scattered pattern (Fig. 3). The abundance in relation to the number of loci on the neo-Y chromosome was also noteworthy compared to autosomes and the neo-X_1_ and neo-X_2_ chromosomes (Fig. 2 and Supplementary Tables S1 and S2). Among the 45 satDNAs families, 39 were located in the neo-Y chromosome and six of them (Esur11, Esur31, Esur37, Esur46, Esur65 and Esur66) through FISH, mapped exclusively to the neo-Y chromosome, however, they were recovered through PCR in both sexes (Fig. 3, Supplementary Tables S1 and S2).

### Male versus female satDNAs abundance

**Figure 4:**
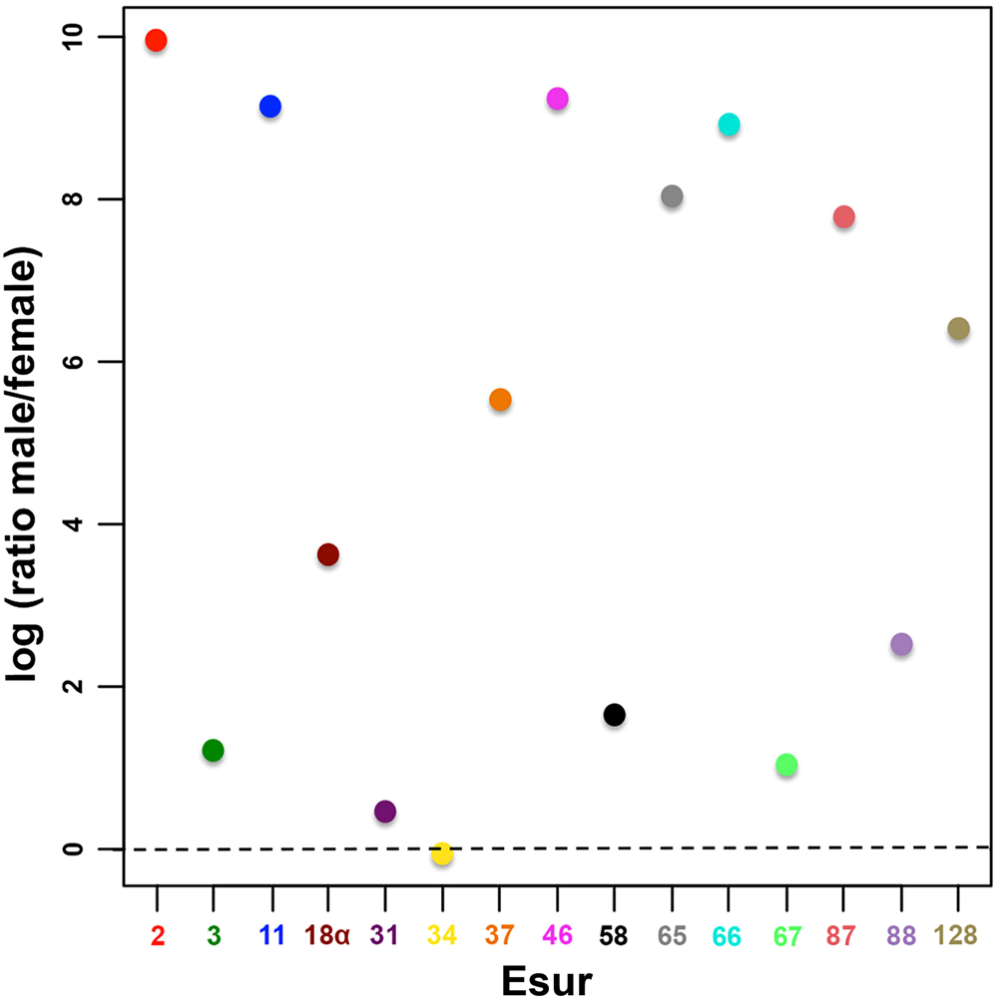
Copy number variation between male *vs* female genomes of 15 satDNAs with monomers >60 bp in *E*. *surinamensis*. The male/female ratio of a relative copy number is shown on a logarithmic scale. The qPCR of male and female genomic DNA was used to calculate the satDNA dose by a ΔCt method of relative quantification (see Supplementary Table S3). Each satDNA family (Esur) is represented by numbers and colors directly in the images. Note that most satDNAs differ significantly between sexes with males having a higher copy number than females for most repeats studied. The statistical significance for difference in copy number between males and females using chi-squared test was highly significant, *p*-value < 0.01 for each satDNA.

The comparative relative abundance of satDNAs between male and female genomes carried out through qPCR clearly show that the satDNAs doses differ significantly between sexes, with males harboring a higher copy number than females for most of repeats studied (Fig. 4, Supplementary Table S3). For example, most remarkable difference were seen in Esur2, Esur11, Esur46, Esur65, Esur66, Esur87 and Esur128, with males showing six to ten times more copies than females (Fig. 4).

## Discussion

### General organization of satDNAs in the genome of *E*. *surinamensis*

Through the graph-based clustering of sequencing reads of the *E*. *surinamensis* genome, followed by complementary analysis, we discovered 45 new satDNAs families. These satDNAs coexist in the *E*. *surinamensis* genome, with variable monomer sizes and nucleotide divergence, accounting for ~14% of the genome (758.8 Mb). It is well known that diverse satDNA families are commonly found in eukaryotic genomes, e.g., in *Olea europea* with six satDNA families^33^, *Tribolium castaneum* with nine^34^, and *Camellia japonica* with four^35^, reaching up to 16 satDNAs in *Drosophila melanogaster* (reviewed by ref. 36) and 62 in the grasshopper *L*. *migratoria* ^30^. Besides the considerable proportion of satDNAs in the genome of *E*. *surinamensis*, it is also remarkable the presence of so many different families in comparison with most eukaryotes. Another interesting feature of *E*. *surinamensis* is that no satDNA family is predominant, which contrasts with what has been observed in several other species, in which a few families prevailed^33–36^.

We could speculate that the accumulation of satDNAs in *E*. *surinamensis* could have been favored by highly rearranged karyotype (2n = 9♂) in comparison with the modal diploid chromosome number in Gryllidae crickets^23^. Alternatively, the high diversity of repetitive DNA itself might facilitate/cause such a complex karyotype rearrangements. It is well known that chromosomal rearrangements may involve highly repetitive DNA sequences, since these could provide sites for karyotype reshuffling without detrimental effects on the integrity of coding sequences^37–39^. In any case, the rearranged karyotype of *E*. *surinamensis* represents a specific environment with limited recombination that could facilitate the rate of homogenization of repetitive DNA, like in multiple sex chromosomes (see below). In addition to chromosome rearrangements, mechanisms involved in the satDNAs evolution could have occurred, such as amplification mediated by rolling-circle replication and reinsertion, unequal crossing-over between DNA repeats from sister chromatids, transposition and gene conversion. These mechanisms have been suggested as possible causes of sequence homogenization within satDNA^1, 8^. The putative involvement of highly rearranged karyotype for satDNA multiplication could be supported by comparative analysis with the related species *Gryllus assimilis* (Gryllidae) with higher diploid number that is ancient for cricket, in which the satDNA analysis revealed the occurrence of only 11 satDNA families (Palacios-Gimenez *et al*., submitted). Considering the genome size (*G*. *assimilis* 2.13 Gb and *E*. *surinamensis* 5.42 Gb) and quantity of satDNA in each species (*G*. *assimilis* 4% and *E*. *surinamensis* 14%) the quantity of satDNAs was increased about 8.9 times in the genome of *E*. *surinamensis*.

We found that most satDNAs are non-homologous, suggesting inefficient homogenization between distinct repeats. The intra-family homogenization is evident, even more for larger satDNAs than smaller ones, and could be directly related to the sequence size and specific chromosomal distribution. Comparing the distribution of the large- and small-sized satDNAs it is evident that the smaller, ranching up to 60 bp, repeats are more dispersed than larger ones (Fig. 2), mostly occurring in all chromosomes of the complement, which could facilitate their sequence divergence.

In our data, the satDNA monomer size was negatively correlated with sequence divergence (*rho* = −0.48, *P* < 0.001) (Supplementary Fig. S3). A similar trend was also observed for three satDNAs of the cave cricket *Dolichopoda schiavazzii* ^40^. This finding disagrees with predictions from computer simulations, which anticipated a direct correlation between the monomer size and the divergence due to low recombination frequency^41^. However, as noted by Bachmann *et al*.^40^, A + T content and sequence complexity, which were not included in these early models, could play a role in facilitating recombination and homogenization of the repeats. We also found a negative correlation between the satDNA monomer size and the number of loci (*rho* = −0.63, *P* < 0.0001) (Supplementary Fig. S3). The distribution of small satDNAs in almost all chromosomes, and remarkably in the neo-Y, could be the result of dispersion through extra-chromosomal circular DNAs, formed via intrastrand recombination, and subsequent reintegration elsewhere in the genome by illegitimate or homologous recombination (reviewed in ref. 42). In addition, the movement of otherwise non-transposable DNA fragments via their capture by transposable elements could represent another possible mechanism of the intragenomic dispersion of tandem repeats^43–47^. What remains is to investigate the exact role played by the monomer size in facilitating satDNA dispersion.

For the large satDNAs, which exhibit in a more chromosome-specific distribution, our combined analysis using cytogenetic and genomic tools revealed a distinct degree of complexity, i.e., simple tandem arrangements and composed units, like α/β repeats for Esur17 and Esur18. satDNAs with composite units have been reported for example in *Chrysolina carnifex* ^48^ *Tribolium brevicornis* ^49^ and primates^50^. Besides the composite units observed for Esur17 and Esur18 we also found cases of overlapping distribution of satDNAs as demonstrated by fiber-FISH for the satDNAs families exclusively located in chromosome pair 1, Esur4, Esur6 and Esur18. It occurred independently of sequence homology. Moreover, we observed other cases of co-localization depending on the chromosome. Overlapping distribution of satDNAs is usually reported for repeats with a remarkable similarity, such as the satellite I and satellite II subfamilies in *Tribolium madens* ^51^ the pBuM-1 and pBuM-2 subfamilies in the *Drosophila buzzatii* species complex^52^ and the psr2 and psr18 subfamilies in *Nasonia vitripennis* ^53^. However, overlapping distribution of non-homologous satDNAs as observed here for *E*. *surinamensis* was reported only in a few cases, e.g., the DBC-150 and pBuM satDNAs family in several *Drosophila* species^54^ the pSc200 and pSC250 families in the rye chromosomes^55^ and the PROsat, PSUchr1sat and PsatDNA satDNAs in two species of hamsters^56^.

The complexity of the evolution of satDNAs identified in the *E*. *surinamensis* genome is reflected by (i) junctions of monomers forming complex units, as explained above and reinforced (ii) by the occurrence of divergent satDNAs, probably originating from a common ancestor, as indicated by the sequence similarity. For example, we found an apparent similarity of monomer satDNA sequences between Esur2 and Esur34 and between Esur3 and Esur58. However, each of these sequences in the constructed NJ tree constitutes separate branches. This allows us to conclude that these sequences belong to closely related satDNAs families with a common ancestor but they diverged during their evolution, including moving to distinct chromosomal regions. Moreover, these sequences differentially amplified which resulted in variation in their copy number, as demonstrated by qPCR experiments.

### Accumulation of satDNAs occurred in the *E*. *surinamensis* neo-Y chromosome

It is well known that the Y or W sex chromosomes accumulate high quantities of distinct classes of repetitive DNAs due to the low frequency of recombination^1, 18, 19, 21, 57, 58^. In some species, it was shown that these repetitive sequences are involved in genetic degeneration, chromatin organization and regulation of expression^36, 57, 59^. This could also be the case in the *E*. *surinamensis* sex chromosomes that remain either unpaired and achiasmatic during male meiosis, suggesting the absence of recombination between them^22, 23^. Regarding satDNAs, more than one family harboring sex chromosomes were reported, for example, in *D*. *melanogaster* ^60^, *Muntiacus muntjac* ^61, 62^, *T*. *castaneum* ^34^, *Rumex acetosa* ^63–65^ and *Silene latifolia* ^66^. However, in comparison with these species the *E*. *surinamensis* neo-Y chromosome harbors the highest diversity of satDNAs documented to date, representing 39 distinct families, with seven being exclusive to this chromosome. Moreover, most satDNAs are enriched in this sex chromosome and constitute multiple loci as shown by FISH. These findings suggest that the neo-Y accumulated satDNAs after its origin, causing its enlargement, in comparison with the neo-X_1_ and neo-X_2_. A similar pattern was reported for the large Y chromosomes of plants that also accumulated repetitive DNAs^65, 66^ in contrast to the small mammalian Y chromosomes^67, 68^.

Our data suggest a complex origin of the neo-sex chromosomes in *E*. *surinamensis*, involving more than two simple translocations anticipated for the Orthoptera neo-X_1_X_2_Y sex chromosomes^22, 23, 25^. The evolution of *E*. *surinamensis* sex chromosomes probably included centric and tandem fusions and inversions, like in *M*. *muntjac* ^61, 62^. The multiple interstitial satDNAs loci in the *E*. *surinamensis* neo-Y could represent remnants of the ancestral centromeric material at chromosome fusions sites, or they are the consequence of massive expansion of DNA repeats that could induce new chromosomal rearrangements.

Although some satDNA families were exclusively mapped by FISH to the neo-Y chromosome, they were also recovered in the female genomic DNA using PCR, indicating their occurrence in other chromosomes, at least in a low copy number. Differential expansion of satDNAs between sexes was corroborated by qPCR. Our results clearly showed that the doses of most satDNAs examined differ significantly between sexes, probably due to differential expansion, with males having on average two to ten times more copies than females. We hypothesize that the differential repeats distribution patterns on the *E*. *surinamensis* sex chromosomes can be explained by a higher rate of colonization and insertion and lower rate of removal of satDNAs in the neo-Y chromosome, similarly to that suggested for the *R*. *acestosa* sex chromosomes^65^. Therefore, these satDNAs lead to compartmentalization of the neo-Y chromosome and a chromosome-wide DNA sequence diversity. In addition, the neo-Y chromosome may exhibit a differentiated long-range chromatin structure/composition compared to autosomes and both neo-X chromosomes. Partially-heterochromatic appearance of the neo-Y chromosome in *E*. *surinamensis* with G + C-rich blocks dispersed in its entire length^25^ supports this view. The involvement of neo-sex chromosomes in increasing of satDNAs quantity and diversity in *E*. *surinamensis* emerges by analysis of *G*. *assimilis* with X0 sex system, which is ancestral for Orthoptera as a whole^22, 23^. In this species only 11 satDNAs families were recovered by the same papiline used here. Among them only eight populate the sex chromosome with discrete bands mainly in terminal region (Palacios-Gimenez *et al*. submitted).

Our data, together with the analysis of sex chromosome that revealed either unpaired and achiasmatic during meiosis^22, 23^ − a common feature for old sex chromosomes^69^ − suggest the occurrence of old neo-sex chromosome system in *E*. *surinamensis*, which contrasts with the described young sex chromosomes^21, 70, 71^. Moreover considerable accumulation of various satDNAs and other repetitive DNA classes in the neo-Y chromosome^25^ reinforce this view, as reported in other species^67, 69^. This could mean that there are no evolutionary strata on the *E*. *surinamensis* sex chromosomes, similar to the strata found in human X chromosome^72^. Theoretically, satDNAs should accumulate more intensively in a region of the Y chromosome corresponding to the ancestral X chromosome, in which recombination was ceased earlier, but no such region was identified in our study, supporting this view.

Our study provides important information concerning composition and evolution of neo-sex chromosomes among crickets, suggesting the involvement of neo-sex chromosomes in amplification and sequence divergence for satDNAs, which generated the highest diversity of satDNAs in sex chromosomes. This data with future analysis using other repetitive sequences and sex related single copy genes will be also relevant to understand precise composition of sex chromosomes, helping in the understanding of sex chromosome evolution, sex determination and possible mechanisms involved in dosage compensation, an issue almost completely unknown in Orthoptera. Moreover to shed light in the evolutionary history of sex chromosomes in crickets it will be informative the analysis of other species with divergent sex systems using similar strategies applied here in a comparative manner.

## Materials and Methods

### Samples, Chromosome Preparations and Genomic DNA Extraction

Males and females of *Eneoptera surinamensis* were collected in the Parque Estadual Edmundo Navarro de Andrade (Rio Claro, SP, Brazil) between May 2013 and March 2014 with the authorization of COTEC (process number 341/2013) and were maintained in captivity until oviposition. Mitotic chromosomes preparations were obtained from embryo neuroblasts using standard procedures described elsewhere^73^. In addition, adult male testes were dissected and fixed in Carnoy’s modified solution (3:1, 100% ethanol: absolute acetic acid). Genomic DNA of adult males and females were extracted from femurs using the phenol/chloroform-based procedure described in Sambrook and Russel^74^.

### Illumina sequencing and graph-based clustering of sequencing reads

Paired-ends sequencing (2 × 300) was applied in libraries prepared as recommended by illumina (illumina Inc., San Diego, CA, USA) using Nextera DNA Library Preparation Kit v3 to one male specimen genomic DNA. Library fragments sizes were in the range from 400 to 600 bp and sequencing was performed using Miseq Sequencing System. The obtained reads were preprocessed to check the quality of the reads with FASTQC^75^ and we did a quality filtering with the FASTX-Toolkit suit^76^. The paired-end reads were also trimmed at 150 nt in length, and then were joined using the “fastq-join” software of the FASTX-Toolkit suit^76^ using default options. Based on illumina sequencing we estimated the G + C content of the whole genome using FastQC High Throughput Sequences QC Report version: 0.11.4 (available at http://www.bioinformatics.babraham.ac.uk/projects/). To search for satDNAs in the *E*. *surinamensis* genome, we carried out a graph-based clustering and assembly of these sequences using the RepeatExplorer^31, 32^. Afterward, we searched for clusters that showed repeat graph density in summary output, which is a typical characteristic of satDNAs families in this approach^31^, and refined this search using Dot plot charts implemented in Dotlet^77^.

### Isolation and sequence analysis of satDNAs

Clusters with high graph density were submitted to the Tandem Repeats Finder (TRF) algorithm^78^ to identify the DNA sequence that maximized the alignment scores between the different monomers that could be defined in tandem. The TRF alignment parameters were 2, 3, 5 for match, mismatch and indels, respectively, and a minimum alignment score of 50 was required for reporting. Additionally, we used the dotplot graphic alignment tool implemented in Dotlet^77^ to identify monomers of the same family and to confirm the tandem organization. The monomers with maximum length were used as the representative copy for each satDNA family, and as the query sequences for further BLAST (http://www.ncbi.nlm.gov/Blast/) and Repbase (http://www.girinst.org/repbase/) searches to check similarity with published sequences. Sequence alignments of satDNAs copies were performed using Muscle^79^ implemented in MEGA5^80^. MEGA5 was also used to estimate nucleotide divergence (*p* distance), A + T content and perform repeat length analysis. The evolutionary relationships among sequences were inferred by neighbor-joining (NJ) trees using the implemented option in MEGA5 and the proportion of nucleotide differences (*p* distance). Satellite alignments are available upon request to the author.

The consensus sequences of each satDNA family was used to design primers with opposite directions (Supplementary Table S4), using the Primer3 software^81^ or manually. In order to verify the presence of satDNAs families in male and female, we performed polymerase chain reactions (PCR). PCRs were carried out using 10× PCR Rxn Buffer, 0.2 mM MgCl_2_, 0.16 mM dNTPs, 2 mM of each primer, 1 U of *Taq* Platinum DNA Polymerase (Invitrogen, San Diego, CA, USA) and 50–100 ng/μl of template DNA. The PCR conditions included an initial denaturation at 94 °C for 5 min and 30 cycles at 94 °C (30 s), 55 °C (30 s), and 72 °C (80 s), plus a final extension at 72 °C for 5 min. The PCR products were visualized on a 1% agarose gel. The monomeric bands were isolated and purified using the Zymoclean™ Gel DNA Recovery Kit (Zymo Research Corp., The Epigenetics Company, USA) according to the manufacturer’s recommendations and then used as source for reamplification.

To check the isolated sequences, the purified PCR products were sequenced in both directions using the service of the Macrogen Inc., and then compared to the consensus sequences obtained by the genomic analysis. The consensus sequences for each satDNAs family can be found in Supplementary Results S1, and sequence alignments are available upon request.

### Probes and fluorescence *in situ* hybridization (FISH)

PCR products for each satDNA family were labeled by nick translation using biotin-14-dATP (Invitrogen) or digoxigenin-11-dUTP (Roche, Mannheim, Germany). SatDNAs with less than 60 bp were labelled directly at the 5′ end with biotin-14 dATP (Sigma-Aldrich, St Louis, MO, USA) during their synthesis. Single or two-color FISH was carried out according to Pinkel *et al*.^82^ with modifications^83^ using mitotic chromosome preparations. Fiber-FISH experiments were conducted as described in de Barros *et al*.^84^ using suspensions of testis cells. The probes that were labeled with digoxigenin-11-dUTP were detected using anti-digoxigenin-rhodamine (Roche) and the probes labeled with biotin-14-dATP were detected using streptavidin conjugated with Alexa Fluor 488 (Invitrogen).

Following FISH, chromosomal preparations were counterstained using 4′,6-diamidine-2′-phenylindole (DAPI) and mounted in VECTASHIELD (Vector, Burlingame, CA, USA). Chromosomes and hybridization signals were observed using an Olympus BX61 fluorescence microscope equipped with appropriate filter sets. Black-and-white images were recorded using a DP71 cooled digital camera. The images were pseudo-colored in blue (chromosomes) and red or green (signals), merged and optimized for brightness and contrast using Adobe Photoshop CS2.

### Quantitative analysis of satDNAs

Quantitative PCR (qPCR) using male and female genomic DNA as templates was used to check the copy number differences between males and females of selected satDNAs, i.e., some of the repetitive families larger than 60 bp length (see primers in Supplementary Table S4). The selected satDNAs were chosen due to their presence on the neo-Y chromosome determined by FISH. The qPCR of male and female genomic DNA was used to calculate the satDNA dose by a ΔCt method of relative quantification^85^. Gene dosage ratios (GDR) of the target satDNAs were compared with a reference gene, 70-kDa heat shock protein (Hsp-70) family using as primers F: 5′-GGTGGTATGACCACTCTTATCAA-3′ and R: 5′-CACTTCATTTTGAGGCACACC-3′ that were designed according to the *Hsp-70* gene from *Locusta migratoria* (accession number AY178988). This gene was used as a reference because there were no differences detected in amplification rates between sexes in *E*. *surinamensis*, suggesting that the *Hsp-70* gene is autosomal and has equal copy number in both sexes. Because we were not sure whether *Hsp-70* is a single-copy gene, the quantitative analysis was a relative comparison of the gene dose between male and female. Quantitative analyses were carried out in MicroAmp® Fast Optical 96-Well Reaction Plate with Barcode (0.1 mL) (Applied Biosystems, Life Technology™, Carlsbad, CA) covered by Optical Adhesive Covers (Applied Biosystems) using the StepOne Real-Time PCR Systems Thermal cycler. qPCR in both the target satDNAs and the reference gene were performed simultaneously in triplicates of three independent samples, i.e. 3 males and 3 females. Each qPCR mixture contained 6.25 μl 2× Go*Taq*® qPCR master mix (Promega, Madison, WI, USA), 0.25 mM of each forward and reverse primers and 30 ng of either male or female genomic DNA, in a final volume of 10 μl. qPCR mixtures without DNA served as negative controls. The cycling conditions were 95 °C for 10 min, 40 cycles of 95 °C for 15 s, and 60 °C for 1 min. Specificity of the PCR products was confirmed by analysis of the dissociation curve. A correlation analysis of GDR means of target satDNAs between males and females were carried out with R statistical software version 3.3.1^86^ and edited using Adobe Photoshop CS2.

### Statistic correlation analysis

We used the PerformanceAnalytics package^87^ implemented in the R statistical software version 3.3.1^86^ to calculate Spearman’s rank correlation coefficients for satDNA monomer size, genome proportion, A + T content, nucleotide divergence and loci number. For the satDNAs showing spread FISH signals (see Supplementary Tables S1 and S2), the total countable loci number was increased by 10 to account for their dispersed profile, but still in a conservative manner to avoid counting the possible existing nonspecific hybridization signals.

## Electronic supplementary material

supplementary information
