# Supplementary material for: High-throughput analysis of the satellitome revealed enormous diversity of satellite DNAs in the neo-Y chromosome of the cricket Eneoptera surinamensis
Source: Sci Rep. 2017 Jul 25;7:6422. doi: 10.1038/s41598-017-06822-8 (PMC5527012; doi:10.1038/s41598-017-06822-8)
Supplement: Supplementary file 1 — supplementary information [file 41598_2017_6822_MOESM1_ESM.pdf]

High-throughput analysis of the satellitome revealed enormous diversity of satellite DNAs in the neo-Y chromosome of the cricket *Eneoptera surinamensis*

Octavio Manuel Palacios-Gimenez[1], Guilherme B Dias[2], Leonardo Gomes de Lima[2], Gustavo CS Kuhn[2], Erica Ramos[3], Cesar Martins[3], Diogo Cavalcanti Cabral-de-Mello[1,*]

[1]UNESP - Univ Estadual Paulista, Instituto de Biociências/IB, Departamento de Biologia, Rio Claro, São Paulo, Brazil

[2]Departamento de Biologia Geral, Universidade Federal de Minas Gerais, Belo Horizonte, MG, Brazil

[3]UNESP - Univ Estadual Paulista, Instituto de Biociências/IB, Departamento de Morfologia, Botucatu, São Paulo, Brazil

Supplementary table S1. Table showing the main characteristics of the satDNAs families < 60 bp observed in the *E. surinamensis* genome.

Cluster	Consensus	Monomer size (bp)	AT%	Genome proportion %	Nucleotide divergence (±sd) %	Reads/ contigs	Chromosomal position (loci number)
Esur 1	TGGAGGAGGA	10	40	1.43	1.9 (±0.8)	27.09	1 (2)<br>2 (1/2*)<br>3 (1)<br>X_1 (2)<br>X_2 (3)<br>Y (11)
Esur 5	TCTCGAGACGG<br>GGGCGACGC	20	25	1.01	6.6 (±2.2)	878.66	1 (2)<br>2 (1)<br>3 (1)<br>X_1 (2)<br>X_2 (3)<br>Y (s)
Esur 12	GGGACGAGAC	10	30	0.486	22.7 (±5.2)	300.95	1 (1)<br>2 (1)<br>3 (1)<br>X_1 (1)<br>X_2 (2)<br>Y (6)
Esur 13	TTGAGACAGGC<br>TTCCGAGAGAC<br>ACC	25	44	0.48	12.7 (±1.9)	346.66	1 (2)<br>2 (1)<br>3 (1)<br>X_1 (3)<br>X_2 (3)<br>Y (s)
Esur 16	GAAGAACTGCG<br>AAGAGATAC	20	65	0.472	20.4 (±4.9)	153.45	1 (1)<br>2 (1)<br>3 (1)<br>X_1 (1)<br>X_2 (3)<br>Y (5)
Esur 21	CCCTCAAGCCGT	31	35.5	0.326	10.6 (±1.4)	248.82	1 (1/2*)

	CGCAACGTCCA<br>CCTCTCAC						2 (1/2*)<br>3 (1)<br>X_1 (3)<br>X_2 (3)<br>Y (s)
Esur 25	GGCA	4	25	0.278	14.1 (±7.4)	116.71	1 (1)<br>2 (1)<br>3 (1)<br>X_1 (3)<br>X_2 (3)<br>Y (s)
Esur 30	GGGAGGAGACG<br>CGGGCGCGC	20	15	0.254	30.2 (±4)	68.875	1 (1)<br>2 (1)<br>3 (1)<br>X_1 (4)<br>X_2 (3)<br>Y (s)
Esur 40	ACTACGCATGG<br>GAGTACACCTG<br>CGGGCAGCGGC<br>TCACTC	39	35.9	0.199	8.8 (±1.1)	286.66	1 (3)<br>2 (1)<br>3 (1)<br>X_1 (3)<br>X_2 (3)<br>Y (s)
Esur 49	CTCCACCTCCTT<br>ACTCCGCCTACT<br>ACCG	28	39.3	0.162	14.6 (±2.9)	123.88	1 (1)<br>2 (1)<br>3 (1)<br>X_1 (3)<br>X_2 (3)<br>Y (s)
Esur 56	GAGTAAAGGGA<br>A GAAGAGGTG	21	52.4	0.142	14 (±1.5)	41.91	1 (3)<br>2 (1)<br>3 (1)<br>X_1 (3)<br>X_2 (3)<br>Y (s)
Esur 68	TAGGCAGG	8	37.5	0.107	14.1 (±2.9)	20.41	2 (1)

							3 (1)<br>X_1 (1)<br>X_2 (1)<br>Y (s)
Esur 70	TTGGGTATTGCA<br>CGTGGCAGATA<br>ATCACATAGCA<br>AA	36	58.3	0.105	25.8 (±3.1)	50.44	1 (1)<br>2 (1)<br>3 (1)<br>X_1 (3)<br>X_2 (3)<br>Y (s)
Esur 76	GGTTAGGTTTTA<br>GGGTA	17	58.8	0.096	27.8 (±2.5)	36.82	1 (1)<br>2 (1)<br>3 (1)<br>X_1 (1)<br>X_2 (2)<br>Y (s)
Esur 79	GAGGAGACGG	10	30	0.092	19 (±4.6)	11.86	1 (1)<br>2 (1)<br>3 (1)<br>X_1 (1)<br>X_2 (1)<br>Y (3)
Esur 91	CGGCGAGGACA<br>TCACAACGCC	21	33.3	0.083	14.4 (±4.8)	357.33	1 (1)<br>2 (1)<br>3 (1)<br>X_1 (3)<br>X_2 (2)<br>Y (4)
Esur 92	GCCCGTGCGCCC<br>AAAGGCAT	20	30	0.081	12.4 (±2.3)	105	1 (s)<br>2 (1)<br>3 (1)<br>X_1 (1)<br>X_2 (2)<br>Y (s)
Esur 113	CACGCGTGTGG<br>ACGGAGA	18	33.3	0.057	6.4 (±1.3)	186.5	1 (s)<br>2 (1)

							3 (1)<br>X_1 (1)<br>X_2 (1)<br>Y (s)
Esur 114	GGAGAGGTGGA<br>GGC	14	28.6	0.057	15.1 (±1.8)	20.72	1 (2)<br>2 (1)<br>3 (1)<br>X_1 (1)<br>X_2 (3)<br>Y (3)
Esur 115	AAAGAGAAGAG<br>GGCGGA	17	47.1	0.057	17.5 (±2.3)	186.5	1 (1)<br>2 (1)<br>3 (1)<br>X_1 (1)<br>X_2 (1)<br>Y (4)
Esur 124	GACGGAGACGG<br>GGAGACGACGG<br>CGCGG	27	22.2	0.053	23.8 (±2.6)	24.78	1 (2)<br>2 (1)<br>3 (1)<br>X_1 (4)<br>X_2 (2)<br>Y (4)
Esur 127	TTTTAGGGTAGG<br>TTAGG	17	58.8	0.051	28.4 (±1.9)	22.06	X_1 (1)<br>X_2 (1)<br>Y (7)
Esur 130	GACGGAGAGAT<br>GTA	14	50	0.051	5.4 (±1.5)	164.5	X_1 (1)<br>X_2 (2)<br>Y (5)
Esur 131	GGCGACAGGGG<br>AAACGGCTTCG<br>AGAGACGACGC<br>GGTGGACACAG<br>TGGCGAGA	52	34.6	0.050	11.8 (±1.5)	81.75	1 (1)<br>2 (1/2*)<br>3 (1)<br>X_1 (3)<br>X_2 (2)<br>Y (3)

(±se)= standard error; *Heteromorphisms.

Supplementary table S2. Table showing the main characteristics of the satDNA families > 60 bp observed in the *E. surinamensis* genome.

Repeat Family	Monomer Size	AT%	Genome Proportion %	Nucleotide Divergence % (±SE)	Reads/ Contigs	Chromosomal position (Loci Number)
Esur2	137	29.2	1.42	10.94 (±1.19)	195.64	1 (1)
						2 (1)
						X_2 (1)
						Y (9)
Esur3	142	33.1	1.19	8.7 (±1.15)	233.08	2 (1)
						X_1 (1)
						X_2 (2)
						Y (7)
Esur4	144	35.42	1.04	11.6 (±1.6)	497.62	1 (1)
Esur6	83	36.15	0.707	15.7 (±2.1)	540.35	1 (1)
Esur11	147	38.1	0.507	5.39 (±0.92)	329.4	Y (9)
Esur17			0.466	9.4 (±1.3)	183.27	αβ 1 (1)
α	163	56.5				αβ X_2 (2)
αβ	317	42				
Esur18						αβ 1 (1)
αβ	517	38.3	0.432	4.3 (±0.6)	623.55	α Y (3)
Esur24	140	67.15	0.289	13.9 (±2)	626.33	1 (2)
						2 (1)
						3 (0/1*)
						X_1 (1)
						X_2 (1)
Esur31	229	46.29	0.254	14.9 (±1.5)	183.22	1 (1)
						Y (3)
Esur34	140	35	0.240	4.07(0.87)	259.33	1 (1)
						2 (1)
						X_1 (1)
						X_2 (1)
						Y (11)
Esur37	234	32.9	0.211	9.91 (±1.66)	273.8	Y (3)
Esur46	181	36.5	0.178	2.21 (±1.05)	770.66	Y (2)
Esur58	141	29.8	0.132	13 (±1.3)	55.22	2 (1)
						X_1 (2)
						X_2 (2)
						Y (5)
Esur65	155	51	0.11	6.5 (±1)	129.64	Y (4)

Esur66	253	33.2	0.109	3.2 (1.1)	117.83	Y (7)
Esur67	60	40	0.108	19.7 (±2.1)	52.15	2 (1)
						Y (1)
Esur82	109	44.04	0.09	6.56 (±1)	167.43	1 (1)
						X_1 (1)
						X_2 (2)
Esur87	82	39	0.087	3.7 (±0.8)	375.33	Y (2)
Esur88	127	44.1	0.086	7.8 (±1.2)	79.57	1 (1)
						Y (2)
Esur96	196	20.9	0.079	10 (±0.8)	24.97	X_2 (1)
Esur128	221	49.8	0.051	7.9 (±1.3)	47.28	Y (2)

(±SE)= standard error; *Heteromorphism.

Supplementary table S3. Results of quantitative PCR and statistical analysis for differences of satDNAs copies between males and females. The GDR *p*-value < 0.01 for each satDNAs copy number differences between males and females using chi-squared test.

		**Target-to-reference gene dose ratio (GDR)**				
**Target**	**Sex***	**GDR Sample I[§]**	**GDR Sample II[§]**	**GDR Sample III[§]**	**GRD mean ±SD**	**GDR ratio male/female**
Esur2	M	4,589,943.414	5,336,423.279	12,461,705.5	7,462,690.73 ± 4,345,333.074	21,157.1555
	F	238.4433767	572.4829607	247.2533517	352.726563 ± 190.3655947	
Esur3	M	3,218,553.361	12,538,601.29	6,864,680.147	7,540,611.598 ± 4,696,646.232	3.37525053
	F	1,192,916.688	2,932,143.585	2,577,208.069	2,234,089.447 ± 918,980.5994	
Esur11	M	1,940,446.966	7,657,517.862	4,982,863.902	4,860,276.243 ± 2,860,506.198	9,366.49006
	F	522.4952048	409.2146383	624.9916148	518.900486 ± 107.9333933	
Esur18 α	M	12,732,367.57	7,245,655.812	5,406,957.028	8,461,660.135 ± 3,811,090.365	37.5797056
	F	318,970.1693	302,083.9154	54,442.96862	225,165.6845 ± 148,091.0891	
Esur31 α	M	23,329.9919	105,843.3597	66,168.62816	65,113.99324 ± 41,266.79241	1.58958397
	F	12,825.7723	35,343.5533	74,719.41985	40,962.91515 ± 31,327.12598	
Esur34	M	103,929.5917	360,856.871	389,427.5245	284,737.9957 ± 157,234.9509	0.9492957
	F	139,544.5605	450,235.4622	310,059.7103	299,946.5777 ± 155,592.1453	
Esur37	M	65,916.2504	371,403.6136	94,695.46835	177,338.4441 ± 168,680.2548	254.764641
	F	538.1566036	683.0776572	867.0277901	696.0873503 ± 164.8211255	
Esur46	M	289,780.2839	1,513,856.053	2,267,163.923	1,356,933.42 ± 997,988.0001	10,341.8971
	F	195.8544264	95.78146359	101.9863084	131.2073995 ± 56.07186099	
Esur58	M	170,329.8368	488,456.9772	216,031.8232	291,606.2124 ± 172,002.429	5.22063215
	F	46,642.28272	21,988.4678	98,938.73395	55,856.49482 ± 39,293.91993	
Esur65	M	2,514,030.864	11,295,314.88	7,181,601.336	6,996,982.359 ± 4,393,552.134	3,120.15107

	F	2,532.122283	2,447.950298	1,747.469315	2,242.513965 ± 430.7820045	
Esur66	M	28,457.96794	131,478.6404	234,376.5249	131,437.7111 ± 102,959.2846	7,510.13156
	F	13.55926978	21.34783014	17.597058	17.50138598 ± 3.895161484	
Esur67	M	849,780.4416	3,608599.544	2,053,082.758	2,170,487.581 ± 1,383,151.702	2.80511074
	F	284,756.551	1,016,353.83	1,020,175.071	773,761.8174 ± 423,495.2932	
Esur87	M	6,271,729.637	1,646,315.361	2,097,688.565	3,338,577.854 ± 2,550,189.989	2,419.44807
	F	1,428.989547	2,030.419591	680.2678782	1,379.892339 ± 676.4135674	
Esur88	M	443,731.0493	2,364,118.033	4,120,315.191	2,309,388.091 ± 1,838,903.005	12.4890365
	F	19,219.50737	173,706.6992	361,813.488	184,913.2315 ± 171,571.7012	
Esur128	M	385,671.1233	2,215,048.739	1,315,818.393	1,305,512.752 ± 914,732.3488	605.944893
	F	934.7247035	3,409.778215	2,119.019259	2,154.507393 ± 1,237.908328	

*M, male; F, female.
§ Mean value (n=3) in three independent samples.
±SD, standard deviation.

Supplementary table S4. List of primers designed and used in this paper.

Repeat family	Oligo name	Sequences
Esur2*	Esur_sat_CL_2_F	5'TGG GAC GGA GCA GCG CTG 3'
	Esur_sat_CL_2_R	5'CCA CGC GCG CCC TAC TCA 3'
Esur3*	Esur_sat_CL_3_F	5'CCT GCA CCA TCA TAA GGA GA 3'
	Esur_sat_CL_3_R	5'GCG GGC CAC CGT GCG CT 3'
Esur4	Esur_sat_CL_4_F	5'TGG CCG GTA GTG TCA AAA AG 3'
	Esur_sat_CL_4_R	5'ACG TTA CAA CGC TAG CCG G 3'
Esur6	Esur_sat_CL_6_F	5'GAG GCG GCC GTC GGC CT 3'
	Esur_sat_CL_6_R	5'GCT ATG ACA TCG CGA CAC CA 3'
Esur11*	Esur_sat_CL_11_F	5'GCA TTC ATT GGT TGC GGC GA 3'
	Esur_sat_CL_11_R	5'TTC GTC TGA CGC CGC AGT A 3'
Esur17α	Esur_sat_CL_17α_F	5'GCC TCC GAA AAC CGT AAA AA 3'
	Esur_sat_CL_17α_R	5'GTG CGC GCA TAT TTG TTC TA 3'
Esur17β	Esur_sat_CL_17β_F	5'CCT GCG TGT ACG CAC ATT 3'
	Esur_sat_CL_17β_R	5'CGT TCT TGT GGT CGC ACT TG 3'
Esur18α*	Esur_sat_CL_18α _F	5'GCA AGG GTA CCT GTG CAA AC 3'
	Esur_sat_CL_18α _R	5'CTA TAT CCG GCA GGG TGG T 3´
Esur18β	Esur_sat_CL_18β_F	5'GTG TTC CCC GGG TGA AAT AA 3´
	Esur_sat_CL_18β_R	5'CCG AGC CGA AAT TTT TCT AA 3'
Esur24	Esur_sat_CL_24_F	5'TGT TTG AAG ATC ATG GAA CAA T 3'
	Esur_sat_CL_24_R	5'TAT GTA GGT TCA AGT ACT TTA C 3'
Esur31α*	Esur_sat_CL_31_F	5'CCT TGC CGG ATA TAG GAA TA 3'
	Esur_sat_CL_31_R	5'GGG TAC CCC CTA AAT AGG G 3'
Esur34*	Esur_sat_CL_34_F	5'GAG CAG GGA GAA GGA GTG AG 3'
	Esur_sat_CL_34_R	5'CCG GTG CAT ACA CCC TCT 3'
Esur37*	Esur_sat_CL_37_F	5'TCA AGT CGT CGC TGC GCC T 3
	Esur_sat_CL_37_R	5'AGG GCG GCG GCG AGT CCT 3'
Esur46*	Esur_sat_CL_46_F	5'CAG CGT AGT TCG CTT GTC AG 3'
	Esur_sat_CL_46_R	5'CCG TTC GCG GGG TAG TCG 3'
Esur58*	Esur_sat_CL_58_F	5'CTG CAC CAG CAT AAG GAG GA 3'
	Esur_sat_CL_58_R	5'GGC GGG CCA CAG TGC CTC 3'
Esur65*	Esur_sat_CL_65_F	5'GCA CGA CTA CGC TTC CTT CT 3'
	Esur_sat_CL_65_R	5'TGG CAA TCT GAG CGG TTG AG 3'
Esur66*	Esur_sat_CL_66_F	5'AGG GTG TGG GCG GTA GTA G 3´'
	Esur_sat_CL_66_R	5'CGT GCC ACC CGC TCC CT 3'
Esur67*	Esur_sat_CL_67_F	5'GGT AGT GTC GGT AGC GAT GT 3'
	Esur_sat_CL_66_R	5'AGA GCC TCA GCC TCC ACC 3'
Esur82	Esur_sat_CL_82_F	5'GGT GTG AGG GAT TTG AGG AG 3'
	Esur_sat_CL_82_R	5'CAT CTC ATC TCT CCC AGC C 3'
Esur87*	Esur_sat_CL_87_F	5'AGC GCT CCG ACT GTA ACT CTT 3'
	Esur_sat_CL_87_R	5'GCT GAC TCA CCT AAT GCT GG 3
Esur88*	Esur_sat_CL_88_F	5' TGC GAG AGC GCT GGA GGA GG 3'
	Esur_sat_CL_88_R	5'ACC ACC ACC ACA ATT ACC AC 3'
Esur96	Esur_sat_CL_96_F	5'CAG CAG CTC GTA CCA CGC CA 3'
	Esur_sat_CL_96_R	5'CTG CTG GAC GCT GCG GCG 3'
Esur128*	Esur_sat_CL_128_F	5'TGA CTC AGA GGA TAA CGG AG 3'
	Esur_sat_CL_128_R	5'CTG CTC TCA CCA CTG TGT CC 3'

*Clusters used in qPCR experiments to test difference in copy number between male and female.

Supplementary Figure S1 contains a Schematic representation of the structurally complete copies and organization of the Esur17-α/β (a) and Esur18-α/β (b) satDNAs found in the *E. surinamensis* genome sequence. The form, size, type of arrays, orientation and consensus sequences are indicated by colors.

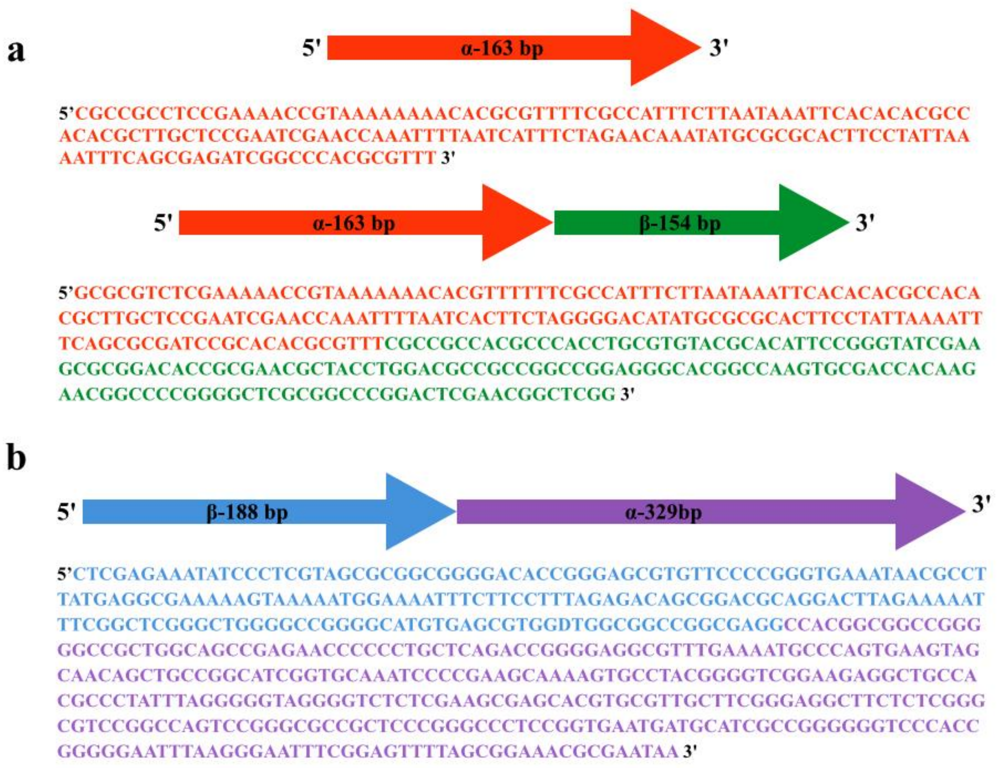

Supplementary Figure S2 includes a NJ tree showing the sequence relationship between Esur2 *vs* Esur34 (a) and Esur3 *vs* Esur58 (b) satDNAs family extracted from the *E. surinamensis* genome. Repeats belonging to the structurally complete copies of each satDNAs family are shown in colors. In the tree, the labels of structurally complete copies correspond to the cluster number (e.g., CL2=Esur2) followed by the contig and copy number (e.g., ctg6-2) representing the repeat analyzed. The nucleotide divergence between each relationship is indicated in the figure. The tree was estimated using the NJ algorithm and the *p*-distance substitution method.

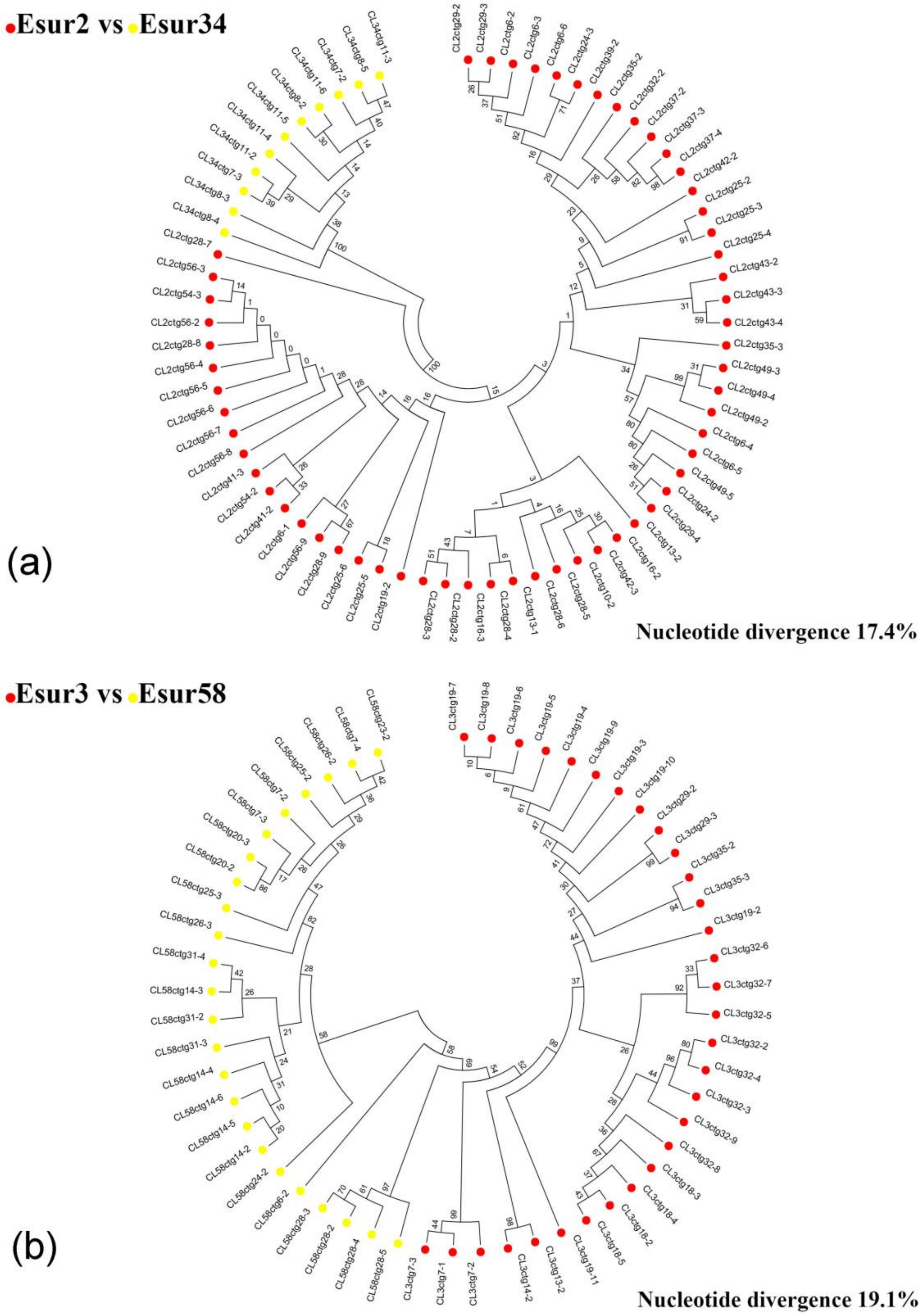

Esur2 vs Esur34
(a)
Nucleotide divergence 17.4%
Esur3 vs Esur58
(b)
Nucleotide divergence 19.1%

Supplementary Figure S3. Plotting showing the negative correlation between (a) monomer size vs nucleotide divergence, and between (b) monomer size vs the total countable loci number of satDNAs. Note in (a) that as the monomer size increases the nucleotide divergence tends to decrease, and in (b) that smaller satDNAs tend to be more scattered than the larger one.

(a)

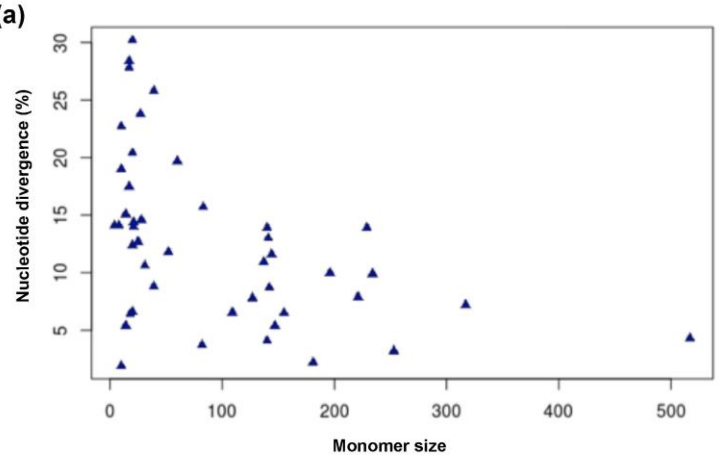

(b)

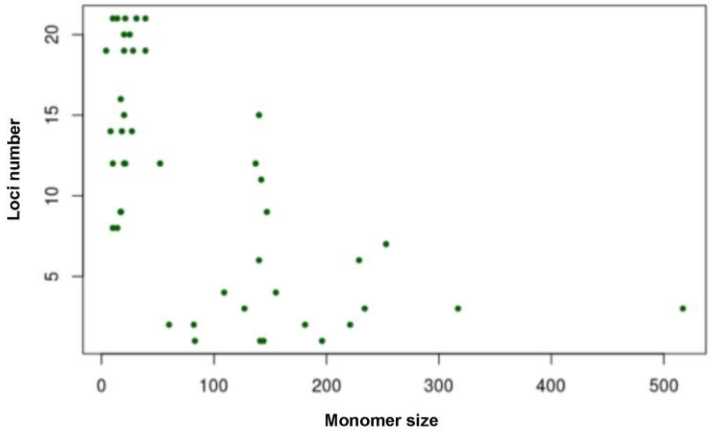

Supplementary Figure S4 shows the Two-color Fiber-FISH for satDNAs exclusively located in chromosome 1, showing the interspersion of Esur4, Esur6 and Esur18-β.

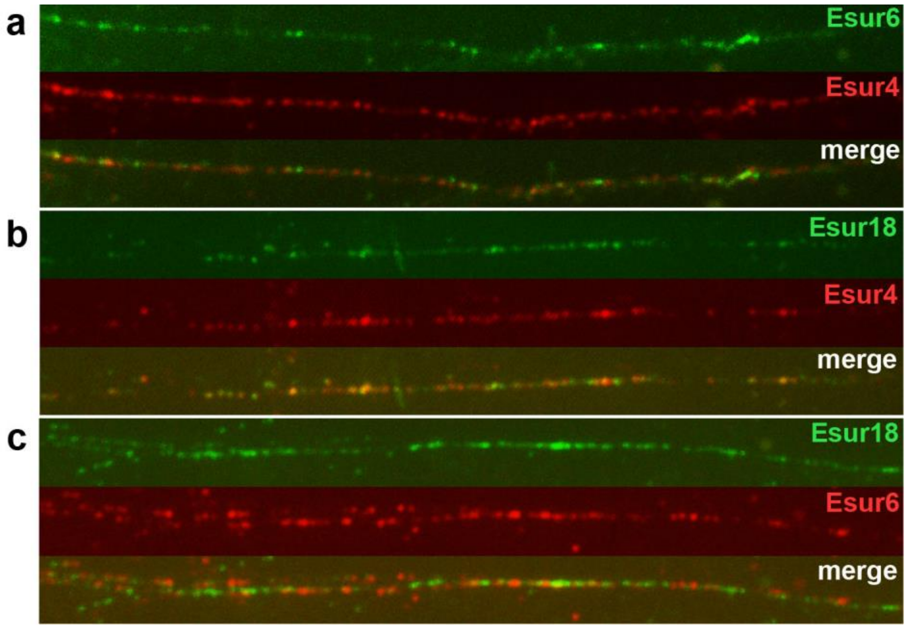

Supplementary Figure S4 FISH on mitotic chromosomes of male embryos showing the location of Esur17-α/β and Esur18-α/β satDNAs. Note the identical location for Esur17-α and Esur17-β and the differential location for Esur18-α and Esur18-β, with the occurrence of Esur18-α in the neo-Y chromosome.

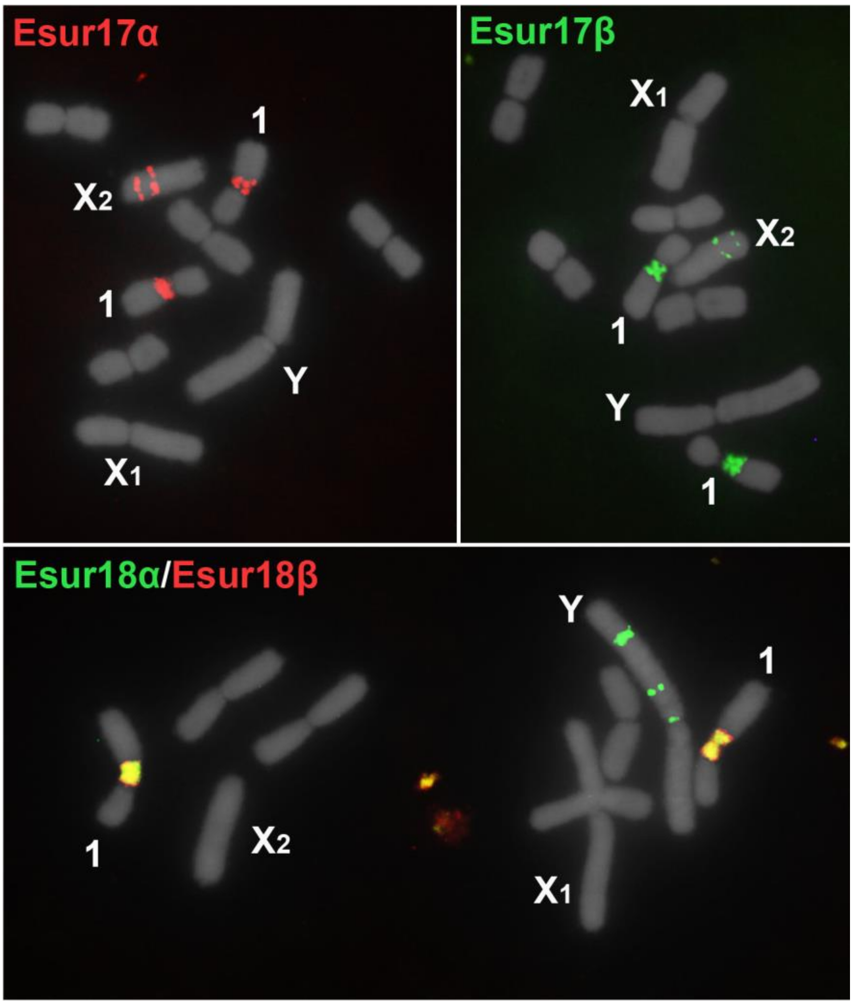

Supplementary results S1. contains monomer consensus sequences recovered from the *E. surinamensis* genome

satDNAs monomer consensus < 60 bp

>Esur1
TGGAGGAGGA

>Esur5
TCTCGAGACGGGGGCGACGC

>Esur12
GGGACGAGAC

>Esur13
TTGAGACAGGCTTCCGAGAGACACC

>Esur16
GAAGAACTGCGAAGAGATAC

>Esur21
CCCTCAAGCCGTCGCAACGTCCACCTCTCAC

>Esur25
GGCA

>Esur30
GGGAGGAGACGCGGGCGCGC

>Esur40
ACTACGCATGGGAGTACACCTGCGGGCAGCGGCTCACTC

>Esur49
CTCCACCTCCTTACTCCGCCTACTACCG

>Esur56
GAGTAAAGGGAA GAAGAGGTG

>Esur68
TAGGCAGG

>Esur70
TTGGGTATTGCACGTGGCAGATAATCACATAGCAAA

>Esur76
GGTTAGGTTTTAGGGTA

>Esur79
GAGGAGACGG

>Esur91
CGGCGAGGACATCACAACGCC

>Esur92
GCCCGTGCGCCCAAAGGCAT

>Esur113
CACGCGTGTGGACGGAGA

>Esur114
GGAGAGGTGGAGGC

>Esur115
AAAGAGAAGAGGGCGGA

>Esur124
GACGGAGACGGGGAGACGACGGCGCGG

>Esur127
TTTTAGGGTAGGTTAGG

>Esur130
GACGGAGAGATGTA

>Esur131
GGCGACAGGGGAAACGGCTTCGAGAGACGACGCGGTGGACACAGTGGCGA
GA

satDNAs monomer consensus > 60 bp

>Esur2_137bp
CGGGGCAGAGAGCGTCGGCGCCGGGAGCAGGGAGGAGTGAGTAGGGCGCG
CGGGGCGGGACGGGGCGGCGCAGGAATGCGCAACAAACACCACTGTATGA
AGGAGGGCGAGAGGCTGTCGCCTGGCCAAAGGCGCAG

>Esur3_142bp
CTCGACAGCCCATTTCCATTCTCCTTATGATGGTGCAGGGCGGGCCACAGTG
CGCTGCCCCCTCCTGCCCCCGCACGCCGCTATCACTAATTTTCCCTGCTCCCG
GTGCCGGCGCACCCCGCCCCGTTGCGCCGACTGGACA

>Esur4_144bp
GGCCGGTAGTGGCAAAAAGGCGGTGAAATGGAGTAACCGCGAGCGCCGGG
CGCCAATCTGAAGGTGGCACTGAGGCCCGGGGACGCGCAGAGGCTTATACC
TCTGCGGGCCCGGGCCACGGATGCCGGCTAACGTTGAAACGTT

>Esur6_83bp
CGCTAAAACATCGCAACCCCAACGGCCCCCGCATACGAAAAGATCGCCGAG
AAACCAGCCCAAGGCGAGGCCGACGGCCGCCT

>Esur11_147bp

CTTCTCTAAGGAGGCCAGGTGCCATCCCGGACGCCGTCGACGGGCAAATGT
TGCCGCCTAGGGGTGGTTGGCGTCACCACACTTCCCTACTGCGGCGTCAGAA
GAAGCATTCATTGGTTGCGGCGACGGGGGATACGGAGTCGGATG
>Esur17α_163bp
CGCCGCCTCCGAAAACCGTAAAAAAAACACGCGTTTTCGCCATTTCTTAATA
AATTCACACACGCCACACGCTTGCTCCGAATCGAACCAAATTTTAATCATTT
CTAGAACAAATATGCGCGCACTTCCTATTAAAATTTCAGCGAGATCGGCCC
ACGCGTTT

Esur17α/β_317
GCGCGTCTCGAAAAACCGTAAAAAAACACGTTTTTTCGCCATTTCTTAATAA
ATTCACACACGCCACACGCTTGCTCCGAATCGAACCAAATTTTAATCACTTC
TAGGGGACATATGCGCGCACTTCCTATTAAAATTTCAGCGCGATCCGCACAC
GCGTTTCGCCGCCACGCCCACCTGCGTGTACGCACATTCCGGGTATCGAAGC
GCGGACACCGCGAACGCTACCTGGACGCCGCCGGCCGGAGGGCACGGCCA
AGTGCGACCACAAGAACGGCCCCGGGGCTCGCGGCCCGGACTCGAACGGCT
CGG

Esur18α/β_517bp
CTCGAGAAATATCCCTCGTAGCGCGGCGGGGACACCGGGAGCGTGTTCCCC
GGGTGAAATAACGCCTTATGAGGCGAAAAAGTAAAAATGGAAAATTTCTTC
CTTTAGAGACAGCGGACGCAGGACTTAGAAAAATTTCGGCTCGGGCTGGGG
CCGGGGCATGTGAGCGTGGDTGGCGGCCGGCGAGGCCACGGCGGCCGGGG
GCCGCTGGCAGCCGAGAACCCCCCTGCTCAGACCGGGGAGGCGTTTGAAAA
TGCCCAGTGAAGTAGCAACAGCTGCCGGCATCGGTGCAAATCCCCGAAGCA
AAAGTGCCTACGGGGTCGGAAGAGGCTGCCACGCCCTATTTAGGGGGTAGG
GGTCTCTCGAAGCGAGCACGTGCGTTGCTTCGGGAGGCTTCTCTCGGGCGTC
CGGCCAGTCCGGGCGCCGCTCCCGGGCCCTCCGGTGAATGATGCATCGCCG
GGGGGTCCCACCGGGGGAATTTAAGGGAATTTCGGAGTTTTAGCGGAAACG
CGAATAA

>Esur24_140bp
GTTTGAAAATCATGGAACAATACAAAGTATAAACCAGCACGTAACTTCAAC
GTAAATCAAATCATACGAAGTAAACAGTTAGGCCTAAAATACACACACAAG
CACTGATCACAATATCAGCTGTAAAGTAACCTACATAT

>Esur31_229bp
CCACGCTGGCTGCAAGGGTACCTGTGCAAACCGGGGAAGCGAAAAAAAAT
GCCCAGTGAAGTAAGCACGAAACCCGGTGTCTGCACAGATTGAAGGTGAAA
AAATGCATTCGGGGCCCGAAGAGGCTGCCACGCCCTATTTAGGGGGGACCA
CCCTGCCGGATATAGGAATAATAATATATATAGGTATGGACCGGCAGGGCC
TCCTCGGCCGAGAAAAGCGTTTGGCT

>Esur34_140bp
GCAGAGGGTGTCCGCACCGGGAGCAGGGAGAAGGAGTGAGTTGGGCGCGC
GGGGCGGGAAGGGGCGGCGCAGGTATGCGCAACATACACCACTGTATGAA
AGAAGGCGAGGGGCTGTCGCATGGCAAAAGGCGTAGCGAG

>Esur37_234bp

CAGAGCGGCACGGTGGGCGATGCGGCGTTCGCGTGCGGTTGGCAGGACTGC
GCGCCGCCCTTCAAGTCGTCGCTGCGCCTCGTGGTGATGCGCGCGCAGAAG
CCGTCGCGCGTGACTGCGCTCGGCTTCGCCACGCTGTCGCTGCCGCTCTTCG
CCGCGGTGAGTGATCACTCGTCTCCACCACTCACTCCTCACGGGGTGACATA
GCAACTAAAAAACCCCGATGCGCGTGCA

>Esur46_281bp
CGCACTCCTCGCGATCAGTGCGCGACGCCAGTGACTGGCCATTGACGCGAC
TACCCCGCGAACGGCAGCGTAGTTCGCTTGTCAGTTGCATCTCTCACACGGA
GAGATTGCTGGTCGAACCGAACGCGACGCGGTGCTCCCCCTCCCGCTTCGC
AGGAAGGAAGTGCGGATCCGCAAACGC

>Esur58_141bp
CTATGGCCCGCCCTGCACCAGCATAAGGAGGATGGAAAGGGGCCTTCGAGT
GTCCAGGCGGCGCAACGGGGCGAGGAGCGCCGGCGCCGGGAGCAGAGAAA
TGACCGAATGCGGCGTGCTGGGGCGGGAGGGGGCAGCGCA

>Esur65_155
AGTGATTTCAAGTGTTTTCAAGTGCTCAAGCAGCAGTCCACCTCGCCGCAGC
GCTCAACCGCTCAGATAGCCAGCACGACTACGCTTCCTTCTGTAAGTCTCAG
TCTAGCTGCAATCTAGCCACCAATGTTATTACTGCAAATGCATTTGCTACC

>Esur66_253bp
GTTGGGGGCGGGCGATGTAGGGGGCGGGCGGCATGAAGGCGCGGGTGGCG
CGAGGGCGCGAGGGTGCGCCCAGCGCGCGGCGCCGATCGCCCGGGACAAA
CGTGGCGCGGCGTTAGTTAAGAGTACAGGAGAGGAAAACAGGGAGCGGGT
GGCACGAGGGTGTGGGCGGTAGAAGGGCAAGAACGCTGGAACGGTAAGGG
CGGTCGAAGGACAAGAAGAGAGCTGTGGAAGAGCAAGGGCGATGTGAGTA
TGC

>Esur_60bp
TGGCGGTAGCGATGTCATCGGCTTCGGTGGTGGTGGCGGTGAAGGCAGTAG
GTCAGGTAA

>Esur82_109bp
GGATTAATGTGGTGTGAGTGAGGAATAGTAAGTGGCTCCGCTTTGGGTAGG
GATGATGGGTGGCGGACTGCGCTCTATGAGCGCTCTTCCGGCTGGGAGAGA
TGAGAGG

>Esur87_82bp
GCCCCGGGCTTCCAGCATTAGGTGAGTCAGCAGCGCTCCGACTGTAACTCTT
CCGTCGGATAAGATGCGCTCAGCGCCTGTG

>Esur88_127bp
TTGTGGTGGTGGTGGTTGCGAGAGCGCTGGAGGAGGAGGAGGATCAGCTGT
CGTGTTGGTGAGAGTATGGTGTTGATAACGCTGAGAGGGTGCAGGTGTTGT
CGTGGATGTCTAACGGCAGTGGTGA

>Esur96_196bp

GCAGCGCCGCGGCGCGCAGGGGCTCACTGGTCCTTGCGCTGGCGGAGGGCG
TGGCGTGTGAGGGGGCGAGGGCATAGCGGCGGGCGAGGCGCCAGAGGAAG
CGGGCGGCGCAGGAGGCGAGGAGGGCGGCCGCCAGCGCCGCCGCCAGCAG
CGCCGCCGCCACGTCCCAGCAGCAGCAGCTCGTACCACGCCAGGC

>Esur128_221bp
GGCATTTCACTATCTGCAACCAATCTACTGAAAACTTGCCAATACCAAGAGT
CCTGTTCACGATCTTTCAGAAGCGACGTCACTTAATAGGAATGTGCGACGCT
CGAGAGGAGGTAGAGAGAGGCGAAGGTCGGAAGAAGACAAGCGCCATCGG
CCGTCCTCACAGCGAGGACACAGTGGTGAGAGCAGTGACTCAGAGGATAAC
GGAGTAAGTTAAG
